# Supplementary material for: Are carbon emissions trading and green financial instruments synergistic? -Comprehensive quantitative research based on content analysis
Source: PLoS One. 2024 Mar 7;19(3):e0298601. doi: 10.1371/journal.pone.0298601 (PMC10919601; doi:10.1371/journal.pone.0298601)
Supplement: S1 Data — (ZIP) [file pone.0298601.s001.zip › Data/地级市Z_F工作报告.zip!/╜¡╬≈/╛┼╜¡╩╨/└┤╫╘╣1⁄2╓┌║┼í╛┬φ┐╦╩2╛▌═°í┐_2005.docx]

各位代表：

我代表市人民政府向大会作政府工作报告，请予审议，并请市政协委员和列席会议的同志提出意见。

２００４年工作回顾

过去的一年是本届政府的开局之年，我们遇到的困难比始料的大，发展的结果比预期的好。在省委、省政府和市委的正确领导下，全市人民深入贯彻党的十六大和十六届四中全会精神，按照“优化投资环境，主攻民营经济”的工作思路，解放思想、与时俱进，认真研究和破解宏观调控中遇到的矛盾和难题，完成或超额完成了年初确定的各项任务，申报中国魅力城市获得成功。预计全市国内生产总值３５６．７６亿元，增长１５．２％；财政收入３５．０８亿元，增长２２．６％；农民人均纯收入２９０１元，净增５２８元，创历史新高；城镇居民人均可支配收入７８１９元，增长１０．８％；社会消费品零售总额超过１００亿元。

一年来，我们主要做了以下几个方面工作：

第一，“五十崛起行动”开局良好。环庐山公路一期通车，九武复线、铜九铁路等六大基础设施项目正在建设。培植利税超亿元支柱财源企业，由２个增加到５个，亚东水泥实现利税２．４亿元，香炉山钨业实现利税１．４亿元，星火有机硅利税过亿元。培育大型市场和物流集团，已有京九、华东建材、国际汽车城、水果批发等７个大型专业市场初具规模，销售额６５．７亿元。倾心实施十大民心工程，农村“五保户”供养补助资金增加１６００万元，集中供养率由１８．８％提高到６２％，城区低保每人每月提标３０或５０元，企业离退休人员平均月增资４１元，近２万名农垦企业职工进入社保；发放特困补助金和救济粮，救助对象６１万人次。新增就业人员５．９万人，下岗再就业２．１万人，困难群体再就业７１００人，超额完成年度目标，城镇登记失业率３．８％。安置三峡移民４００多人，我市被树为全国三峡移民工作先进典型；安置湖区、库区、深山移民７４５０人。新建改造乡村公路１３３７公里。改造农村中小学危房１６．４万平方米，资助贫困学生９．８万人，减免学杂费９７４万元。综合治理水土流失３２万亩；解决１．３万人的饮水困难；建成环庐山生物防火林带８４公里，组建森林消防队１２支；建成气象、森林防火、地质、地震等防灾减灾体系，日元贷款防洪工程进展顺利；安全生产四级管理网络初步形成。空气、噪声、饮用水源、水质监测系统进一步完善。抗洪警示教育基地、城市森林公园基本建成；疾控中心、急救中心、地震监测中心、妇保大楼、廉租房首期工程、少儿活动中心、老年公寓建设开始启动。

第二，主攻民营经济成效显著。从机制层面优化政务环境，加强政府自身建设，纠正部门和行业不正之风。新增１３个部门进驻行政服务中心，取消和调整８７个行政审批事项。市直５０个部门和单位公开服务承诺。处理外商投诉事项件件有着落。民营企业新增１６９８户，增加注册资金１９．４亿元，销售收入２２３．４亿元，增长４１．６％，上缴税金１１．５８亿元，净增３．９５亿元，对财政增长的贡献率达６０．８％。新增税收超千万元企业４个，民营企业创汇６８００万美元，二产比例达６２．２％，由三产为主逐步转到以二产为主，我市被评为全省发展民营经济先进单位。经贸、统计、审计、工商、国税、地税、物价、质监、药监等部门加大了规范市场经济秩序的整治力度。

第三，贯彻宏观调控破解发展难题。认真落实国家宏观调控政策，面对发展出现的新情况 ，我们采取了“存量换增量，开放抢商机，调整促优化，环境造绿洲”的举措破解难题。一是破解经济可持续增长乏力难题。通过向上争资、招商引资、激活民资、银行融资等多种手段，重点推进投资超亿元的项目建设。全社会固定资产投资１４０亿元，增长１７．８％。大力实施《工业崛起纲要》，狠抓大项目建设，完成规模以上工业增加值７３．１４亿元，增长３３．４％；销售收入２６１．４亿元，增长４８．８％；利税１７．９６亿元，增长４５．５％，三项指标实现了三年翻番。２２个重点项目完成投资５１．２亿元，抱子石水电站第二台发电机组、赛得利年产６万吨粘胶短丝、环宇水泥、富达印染等项目竣工投产。九江石化销售收入突破百亿元大关。完成《沿江产业规划》论证，１３个工业园区完成基础设施投资１９．７亿元，入园企业６３９家，安置就业１０万人。二是破解农民增收难题。全面贯彻中央“三补一降”惠农政策，核发补贴资金７０６１万元，核减农业税７５０９万元，农业和农村经济出现了转折性变化。粮食播面３５４．５万亩，增长１７％，粮食总产量１１３．４万吨，增长２８．１％。建立了７１个省级无公害农产品生产示范基地。修水神茶、庐山云雾茶获得原产地保护认证。水产品总量２７万吨。县域经济发展势头强劲，县级财政收入总量突破２０亿元，浔阳、瑞昌、修水三个县（市、区）财政收入过两亿元，９个县（区）超亿元，瑞昌、德安、彭泽分别获得全省经济发展、工业发展、农业发展先进县，争先进位取得了明显成效。三是破解生产要素约束难题。加强调度，多方协调，积极争取有关部门的支持，能源、资金流、人流、物流大幅增长，经济景气指数明显攀升。金融机构本外币各项存、贷款余额比年初分别增加３８．９亿元和４０亿元。消耗标准煤增长１２％，石油用量增长８％；统配用电量突破３０亿度。全社会客运量４７５３万人次，增长１８．６％，货运总量２５８２万吨，增长２０．１％，尤其是水路货运大幅增长，增幅达６２．３％。四是破解城建和园区开发资金不足难题。积极推进国土资源管理方式的转变，土地市场整顿通过国家有关部门的验收，新增储备土地３８４０亩，市本级土地交易额达１０亿元，实现土地收益５亿元。首次运用ＢＯＴ方式建设老鹳塘污水处理厂，市自来水公司实行股份制改造，鄱湖水产是全省第一家规范设立的水产行业股份公司，庐山旅游核心企业已经注册。

第四，创新体制机制提升开放水平。全市１７４家国有企业完成产权制度改革，其中市直完成五棉公司、三棉公司、钢铁公司等１０家企业改制，涉及资产２３亿元，处理债务２１．９亿元，１．５万离退休人员纳入社会化管理。九江民航顺利移交江西机场集团公司；粮食、交通、港务、农垦、物资等下属企业改制稳步推进。深化农业服务体系改革，林权改革全面铺开。出口加工区的申报取得了实质性进展，与美国路易维尔市结成国际友城，成功举办了赣台经贸洽谈会等系列招商活动。新批外资项目１８１个，增长１０１％；实际利用外资３．０８亿美元，增长２２．７％（同口径）；实际利用内资１５９亿元，增长２５．８％。外贸出口８６７８．４万美元，增长７３．６％，外贸国际集装箱吞吐量超２万标箱，比上年翻一番。接待境外游客６．０４万人次，增长５４．６％，旅游外汇收入１６６２．６万美元，增长６９．７％；接待国内游客６６８万人次，增长１０．４％，国内旅游收入４２．３２亿元，增长１１．１％。庐山申报世界地质公园获得成功。检验检疫、海关、外汇管理等驻市机构和口岸、外事、侨务、对台事务、驻外机构、民间商会在对外开放中发挥了积极作用。

第五，社会各项事业协调发展。完善城市功能，两湖引水置换工程投入使用，三水厂二期日产１０万吨主体工程完工。浔南大道、长江大道、金凤路、青年南路等城区主干道建成通车。八里湖堤路结合工程、青年南路延伸段、滨江生态园开工建设。制订了老龙开河疏浚工程规划方案。完成长虹大道街景的高标准改造。锁江楼公园建成开放。重建了锁江楼、娘娘亭。编制了１０个专项规划和２０个控制性详规，规划覆盖率达９０％。坚决取缔非法营运，集中整治城市重点地段的“三占”、“三乱”现象。人口出生率控制在８．０９‰以下。争取国家、省级科技项目７２个。高中阶段毛入学率达５５．７％，新增民办学校６３所，九江学院在校生突破３万人。建立健全疾病控制和医疗救治体系，全市乡镇卫生院上划县管。庐山国际高尔夫球场投入使用，全民健身运动蓬勃开展，张丽萍在残奥会上打破世界纪录。邮电业务总量１３．８亿元，增长１５％，计算机网络和移动电话用户实现了同步增长。《九江市志》正式出版，《九江年鉴》首次发行，完成了第三次文物普查，乡村有线电视覆盖面扩大。村落社区建设走在全省前列。信访、档案、残联、民族宗教、老龄事业又迈上新台阶。深入开展严打整治，社会治安综合治理得到加强。国防教育和后备力量建设又有新起色，驻浔解放军、武警官兵在急难险重的关键时刻发挥了重要作用。

第六，努力塑造责任型政府形象。切实提高领导抓发展的能力和抓落实的能力。对“五十崛起行动”方案和《工业崛起纲要》中的目标任务，明确责任领导、责任单位和完成时限，年初公开承诺，全过程督查，年终向全市人民汇报。以贯彻《行政许可法》为契机，加大法律知识培训力度，提高公务员依法行政能力，规范行政行为。确认市直执法主体资格４５个，办结行政复议９２件，办理人大代表建议、政协委员提案７８２件。全面开展“百户企业法人评行风“活动。在全省率先建立政府信息公开制度，设立新闻发言人，在“中国九江”网站上定期发布政务信息。加强政府廉政建设，规范工程招投标，扩大政府采购范围，推进药品招标采购，从源头上防止腐败。取消不合理的涉农收费和教育收费，解决征地补偿和拖欠农民工工资问题。专项治理领导干部收受“红包”现象。全面清理非税收入，严格执行收支两条线制度。

各位代表，过去一年所取得的成绩，是省委、省政府和市委正确领导的结果，全市人民和所有在浔工作的同志们、朋友们付出了辛勤的劳动，作出了巨大贡献。对此，我谨代表市人民政府，向所有参与、关心和支持九江发展的同志们、朋友们表示衷心的感谢！

在肯定成绩的同时，我们也清醒地认识到，当前我市经济社会生活中还存在着一些困难和问题：体制和机制的深层次矛盾尚未得到根本解决；经济发展环境还存在一些不尽人意的地方；在加快发展中，资金、土地、电力、人才不足的矛盾日益突出；市本级财政增收后劲不足；就业与再就业形势依然严峻；农民持续增收难度大，部分城镇居民生活还较困难；影响社会稳定的因素依然存在。对于前进中的问题，我们将坚持用改革和发展的办法，努力克服和解决。

２００５年主要任务

２００５年是“十五”计划的最后一年，也是实现九江在江西率先崛起的关键之年。按照市委“狠抓重大项目，谋求科学发展”的思路，今年政府工作的总体要求是：坚持用科学发展观统领全局，创新思维在调控中抢抓机遇，创新理念在实践中破解难题，创新机制在统筹中谋求协调，发挥开放带动、港口带动、中心城市带动的作用，全力推进１００个重大项目、１００个示范村庄、１００件惠民实事的“三百工程”，实现经济和社会持续和谐全面发展，加快九江在江西率先崛起步伐。

今年我市经济和社会发展的主要预期目标是：完成国内生产总值４２０亿元，增长１５％；财政总收入４０．３４亿元，增长１５％；地方一般预算收入２１．２５亿元，增长１３％。全社会固定资产投资１７５亿元，增长２５％；实际利用外资３．２亿美元；实际引进内资１２６亿元（５００万元以上的工业项目）；外贸出口１．１５亿美元。社会消费品零售总额增长１４％，农民人均纯收入增长１０％，城镇居民人均可支配收入增长１０％，城镇登记失业率控制在４．５％以内，人口自然增长率控制在８‰以内。

为了实现上述目标，今年将重点做好七个方面的工作：

一、狠抓重大项目建设，促进全市经济持续快速健康发展

抓重大项目就是抓发展，抓重大项目就是抓后劲。今年确定亿元以上投资或有重大影响、特殊意义的项目１００个，其中工业项目５０个，一、三产业和基础设施项目５０个，总投资１２０亿元。建立健全领导包干责任制、重大项目调度制、现场办公制。从项目引进到竣工投产，专人负责、全程跟踪、包干到底。对引进和实施过程中遇到的困难和问题，及时沟通协调，特事特办，现场解决。项目任务量化细化，责任到人，从严考核。

对已开工的庐山国际会议中心、铜九铁路、沿江原油管道、星火有机硅扩改、亚泥三期、九江石化延迟焦化装置和柴油加氢精制装置等项目全力以赴搞好服务，力促早日竣工。配合做好武（宁）吉（安）高速公路的开工建设。争取昌河发动机项目、轿车项目和３Ｔ光电投影机生产线、数字背投生产线尽快建成投产。抓紧凤竹棉纺、巨石池窑拉丝、恒生科技、台资科技园等项目开工建设，紧跟荷兰ＲＤＭ、远东纺织城、台湾旺旺、台湾中正等一批有意向性的大项目，促其签约进驻。继续争取帽子山核电站、长江二桥、西气东输工程江西段的立项，九景衢铁路列入国家“十一五”规划。

进一步实施《工业崛起纲要》，把培植利税过亿元的企业作为重点，支持工业六大支柱产业做大做强，围绕九江石化、昌河汽车、亚东水泥、赛得利化纤、３Ｔ光电等大项目发展上下游产品，延长产业链，形成产业集群，力争利税过亿元企业达７户。深化国企改革，对啤酒厂、制氧机厂、整流器厂、轻机厂等１０家国有企业，通过引进大财团、大公司并购，实施改制重组。着力培植财源，发展高利税、高附加值产业，扭转市本级财源后劲不足的状况，做大财政蛋糕。做好九江的产业规划，推出一批国家鼓励的基础设施、农业、文化、卫生等大项目，争取国家和省重点扶持。

二、搞好示范村庄建设，努力提高农业综合生产能力，积极探索解决“三农”问题新途径

从落实科学发展观、统筹城乡发展的高度，顺应农民要求改变农村落后现状和追求健康文明生活方式的强烈愿望，结合九江既有山区、湖区，又有城郊区的特点，遵循“分类指导，突出特色，设施配套，协调发展”的原则，从制订规划、扶持产业、完善设施、倡导文明入手，搞好１００个示范村庄建设，加快完善村庄道路、农村电网、生活用水、疾病防治、广播电视等方面的公共设施，每村扶持一个特色的种养加产业，逐步改善农民的生活环境，提升农民的生活质量。今年示范村庄要有看得见的变化，三年要有显著变化。

做大龙头、做响品牌，推动农业产业化进程，促进农业产业结构的调整。从资金、土地、政策、服务等方面加大对龙头企业的帮扶力度，农业专项资金向种养加大户和龙头企业倾斜，壮大鄱湖水产、美庐乳业、上好佳食品、绿冬丝科等企业，培育１－２个能带动１０万农户的大型龙头。做响“鄱湖”大米、“芦花”彭泽鲫、“庐山云雾”茶、“独一枝”山药、“春赐福”罐头、“仙客来”食用菌等品牌，支持珍珠、石材等产品尽快注册商标，培育新的品牌。

大力发展开放型农业，积极鼓励民间资本、工商资本和外商资本下乡，加快向农业开发、农副产品加工和流通领域转移。用资源吸引资本，推行农业、林业、水利等产权制度改革，通过产权买断来吸引社会资本，进行果业、油茶、“三竹”开发和水面网箱养殖。用品牌吸引资本，对已形成规模、具有影响力的珍珠、青虾、黄姜、茶叶、蔬菜、早熟梨等，精心包装重点推介。用市场吸引资本，对市场前景好、销路广、效益高的农业项目，降低准入门槛，鼓励各种资本参与开发经营。扶持印尼金光、新时代油脂、松安林化、正华木业、金绿洲生物工程、立信园艺等外向型企业做大做强。

建立农业增效、农民增收的长效机制，提高农业综合生产能力。严格执行中央１号文件精神，贯彻落实好省委、省政府的惠农政策，加大对传统农业的扶持力度，全部免征农业税，继续做好基本农田保护工作，确保粮、棉、油稳定增长，在扩大面积、提高单产和加工转化等方面下功夫，实现农民持续增收。加大农田水利等基础设施建设力度，重点抓好４座中型、２９座小（一）型病险水库除险加固，争取用３－４年时间消除全市病险水库隐患。认真做好老建扶贫和深山库区移民搬迁工作，相关部门要通力协作，实现搬迁８０００人。健全农业社会化服务体系，继续实施“阳光工程”，确保全年培训农民工２万人，提高他们的就业能力和收入水平，使农民不仅“出得去”，而且能在城市“融得进、稳得住、挣得多”。建立健全市场信息服务网络，为农户提供产前、产中、产后服务。成立产业协会，发展农村合作经济组织，引导专业户以多种形式实行联合。加大农技推广力度，推进农业标准化建设，全面普及良种良法，鼓励科技人员为农户提供有偿承包服务。

三、切实办好惠民实事，努力构建和谐平安九江

让老百姓都能分享改革开放的成果，让困难群体的生活有明显改善，以人为本、执政为民，共建和谐、共保平安，今年要着力为全市人民办好１００件实事。

办实事要从解决老百姓衣、食、住、行做起。继续实施“一扩二帮三改四建五助”等民心工程，加大县乡公路改造力度，今年新建、改造乡村公路１０００公里，力争年内所有乡镇通油路或水泥路，每年硬化通村公路６００公里，到２００７年实现省里提出的目标。解决１．６万人的饮水困难，启动“安全饮水”工程，完成安全饮水、改水、改厕各１万户。加大对义务教育的投入，分三年解决农村中小学学生自带课桌椅问题。加快农村教育发展步伐，力争小学入学率达９９％，初中缀学率控制在２％以内，高中毛入学率达６０％。健全资助家庭经济困难学生就学制度，继续推进职业教育和民办教育，全市职业学校招生规模达２．５万人，民办高中在校生突破３万人，高校在校生突破６万人。建成劳动力中心市场，落实好各项就业优惠政策，新增城镇就业５万人，下岗失业人员再就业１．７５万人，发放小额贷款２０００万元，培训１．６万人，跨省劳务输出５７万人。加强劳动监察，切实解决拖欠农民工工资问题。完善公共卫生体系，搞好新型农村合作医疗制度试点，免费治疗血吸虫病患者２０００例，白内障复明手术１６００例。强化食品、药品安全监管。积极发展体育产业，实现群众体育与竞技体育协调发展。深化人口与计划生育综合改革，严格控制性别比，稳定低生育水平。全面启动工业污染源达标治理工程，完成大气、水、噪音自动监测点建设，加强资源开发与保护。抓好省市级制造业信息化应用示范企业和培训基地建设，办好鄱阳湖水产科技园和武宁民营科技园区，建设县（市、区）地震监测工作网络。启动市博物馆建设和农村无线数字电视有效覆盖工程。

办实事要从帮扶困难群体做起。继续落实“两个确保”和“一个低保”三条保障线，探索困难群体长效帮扶机制，逐步建立与经济社会发展水平相适应、覆盖城乡的社会救助体系，使救助工作走上规范化、制度化、法制化轨道。进一步完善城市最低生活保障制度、农村特困群众救助制度，把农村无法维持最低生活水平的家庭纳入救助范围。逐步建立农村医疗救助制度，使特困人员享受医疗救助。加快市救助站的搬迁，提高农村“五保”和城镇“三无”对象集中供养率。抓好残疾人就业和扶贫工作，帮助１０００名残疾人脱贫。

办实事要从营造和谐平安环境做起。严格信访工作目标管理责任制，坚持“分级负责、归口办理”的原则，健全包案处理、定期处理等信访制度，完善信访网络，加大信息排查，及时掌握信访动态，把矛盾化解在萌芽状态。落实安全生产责任制，坚持不懈抓好安全生产，防止重特大事故发生。加强政法队伍建设，抓好社会治安综合治理，继续保持严打高压态势，深入开展打黑除恶和查禁“毒赌色”各类违法犯罪活动，全力维护社会政治稳定和治安秩序平稳。在全市开展“和谐平安九江”创建活动，加强精神文明建设，倡导守法诚信、邻里和睦、互帮互助的社会新风尚。争创全国双拥模范城“三连冠”和全国社区建设示范城市。

四、强力实施开放带动，提高利用外资的质量和水平

进一步发挥通江达海的地理位置优势，对接大公司，构筑大平台，开展大活动，发展大旅游，把我市建设成对接“长三角”的重要基地，形成大开放的格局。

对接大公司。突出重大项目招商，坚持“走出去、请进来”并举，对外敞开门户，主动出击；对内全面放开，激活民资。顺应国际产业转移的新趋势，紧盯大客商，认真分析世界５００强和国内百强企业的产业升级动态，有针对性地接触国际国内一流公司。突出专业招商、专题招商和产业链式招商。以昌九工业走廊、沿江产业带为依托，花大本钱、下大力气对接国内外一流公司，力争引进世界级品牌，借梯“上楼”。对有投资意向的香港世贸、澳大利亚白马等大公司、大财团，抢先接触，紧盯不放。争取在港口码头、特种材料、大型物流、能源建设等领域引进、对接一流公司有重大进展。继续扶持壮大民营经济，激发民间投资和创业热情，建立“互信、共赢、相依”的新型银企关系，加大对民营企业支持力度，力争民营企业上缴税金突破１５亿元。

构筑大平台。整合沿江沿路工业园区资源，加强产业配套，增强产业集聚度和关联度，壮大产业集群。抓住申报建设出口加工区的契机，以品牌招商，以招商建设新平台，提升对外开放层次。促成九江机场早日复航。继续申报九江开发区为国家级经济技术开发区，改进工业园区的考核办法，重点考核投资密度、就业人数、利税三大指标，做实县区工业园。做强骨干出口企业，提高产品档次，扩大总量规模，帮助更多私营企业获得出口经营权，完善出口通关的各项设施，为外贸出口企业提供高效便捷服务，鼓励本地产品、机电产品出口和加工贸易。

开展大活动。坚持“政府搭台、企业唱戏”，精心筹备赣台经贸洽谈会、沿江产业推介会。同时，积极参加省里组织的各项招商活动，发挥商会在招商引资中的重要作用。继续建立招商引资目标责任制、质量考评制和激励机制，提高招商引资的履约率、进资率和开工投产率。

发展大旅游。抓紧出台《旅游发展总体规划实施办法》。加快庐山旅游集团公司的上市步伐，创建全国文明旅游景区。理顺云居山－柘林湖的管理体制，力争申报国家重点风景名胜区成功。积极配合国家开展“红色旅游年”活动，整合旅游资源，打造红色旅游精品线路。宣传促销“中国水岸、江南九江”旅游品牌，举办“百强旅行社老总共青高尔夫”邀请赛，在新加坡、韩国、日本、南非召开“中国·九江旅游”推介会，大力拓展境外客源市场，力争旅游外汇收入增长２０％，旅游总收入增长８％。

五、着力实施中心城市带动，提升城市综合竞争力

把市委提出的“环绕八里湖建设新九江”作为今年城市建设的重中之重，进一步拉开城市框架，完善城市功能，打造城市特色，增强中心城市的集聚力、辐射力。

启动申报国家级历史文化名城和省级园林城市、卫生城市。完成城市总体规划修编、市域城镇体系规划、环八里湖周边的控规、历史文化名城保护规划、都市圈规划和中心城周边重点镇控规。突出规划范围内的绿地保护、生态保护和水流域保护，力争中心城区详规覆盖率达１００％，县（市、区）城区控规覆盖率达８０％，村庄规划控制率达３０％。

实施拓展工程，以开发八里湖为突破口，采取国际招标，高标准规划，高起点启动重要基础设施和带动项目，真正做到规划与建设齐头并进。同时，加快濂溪组团及综合工业园区路网建设，实施前进西路、长虹西路延伸线，德化东路等路网工程。狠抓特色工程，搞好城市森林公园的设施配套，抓好滨江路、十里大道、九瑞大道的景观改造，完善荷花垄区域、长江大道与高速公路接入口的美化，新建会展大楼、莲花公园，改造南湖公园，完成城区３０万平方米的绿化。打造“记忆”工程，保护好九江的文物和山水资源，挖掘整理民俗文化，重建琵琶亭，疏浚老龙开河，开放浪井，办好锁江楼名人书画展，把九江的“根”留住。建好安康工程，加快老鹳塘污水处理厂、廉租房小区、外国语学校、人防指挥中心、赣北地震监测中心、日元贷款防洪工程、少儿活动中心、体育中心、疾病控制中心等续建工程建设进度，做好老城区边街小巷的硬化、亮化、排水工作。加强县城和中心集镇建设，突出地方特色，不断完善县城和集镇的功能设施，增强县城和中心集镇吸纳人口、带动经济的能力。

完善城市管理执法机制，努力形成统一领导、分级负责、条块结合、权责分明的城管新格局。加强城管综合执法，抓好环境卫生、占道经营、户外广告等专项整治。建立违法建筑责任追究制度，按照“守土有责”的原则，明确整治和管理责任，发挥职能部门作用，加大执法监督力度。全面开展交通秩序整治，规范各类车辆营运，确保城区道路畅通。加强社区建设，完善“自我管理、自我服务、自我约束、自我发展”的机制，给予社区更多的政策和财力支持，切实扭转“变重视为重负”的状况，增强社区服务功能。

六、加快实施港口带动，推动昌九工业走廊建设和沿江开发

港口的辉煌，历来见证九江的辉煌。充分发挥港口优势，突出港口在九江经济和社会发展中的重要地位，实施“港口带动”，以港促城，以港兴业。

实施工港联动。根据“借助昌九路崛起工业廊，沿江产业带经济挑大梁”的发展构架，认真搞好“Ｔ”型经济带的产业规划和设施规划，注重与港区规划相衔接，突出抓好沿江沿路基础设施配套建设，水、电、路、通讯等超前设计、分步到位。制定统一的开发政策，强化协调机制，重点解决跨行业、跨区域的各种问题，市县联动，推动开发进程，力争今年每县都有１－２个亿元以上项目落户沿江、沿路工业园。

实施港贸联动。依托港区加快区域性物流中心建设，新建粮食交易、花卉苗木、鄱阳湖水产、旧货交易市场。开拓现代物流业招商新领域，吸引国内外知名物流企业在我市设立物流基地、配供中心，努力发展现代储运、物资配送、多式联运等第三方物流服务。加大各类中心批发市场、专业市场等物流载体的招商，力促沃尔玛购物广场落户。大力发展就业容量大的传统服务业和信息、中介等新兴服务业，鼓励发展连锁经营、电子商务等现代营销和服务方式，进一步提升九江商贸流通业的档次和水平。

实施港路联动。京九铁路、昌九高速、九景高速对九江经济拉动作用日益显现，发挥黄金水道和陆路便捷的综合效应，是九江能够将区位优势转化为经济优势的关键。加大沿江港口岸线资源的保护力度，搞好码头的规划布局，对适合建深水码头的长江岸线，尽快出台控制性详规。尽早启动湖口国际集装箱码头建设，扩大港口吞吐能力。加强与海内外的物流商贸联系，争取更多的外轮入港，与大型物流集团和水运企业联络洽谈，高起点扩改九江港。制订好出口加工区建设的实施方案，确保江西唯一的出口加工区顺利实施。

七、加强政府自身建设，不断提高依法行政能力

宏观调控的新形势，经济发展的新任务，对政府工作提出了更高要求，迫切需要我们以人为本，以民为先，围绕“开拓、透明、诚信、高效”目标，不断强化自身建设。

以开拓促创新。扎实开展政府系统的先进性教育活动，推动政府系统创新思维、创新理念和创新机制，破解前进中的难题。在用地上向内挖潜，注重盘活存量，集约经营，推行“无地招商”、“零地技改”。多渠道融通资金，既要争取银行的信贷支持，又要在强化招商和激活民资上做文章，加大项目招商力度，加快重大基础设施引入ＢＯＴ、ＴＯＴ方式建设步伐。在能源上，搞好规划，制定好应急预案，加大技改投入，提高能源利用率。在招商上，不搞压地价比优惠，注重环境质量和服务质量。

以透明促规范。贯彻实施国务院《全面推进依法行政实施纲要》，积极做好“四五”普法的考核验收工作。健全政府的决策机制、工作推进机制、监督机制、责任追究机制和长效管理机制，政府的行为必须规范在法律法规的范围之内，依法依规行使权力，在实施过程中要建章立制，加强监察、审计监督，力戒行政行为的随意性，从机制上防止腐败，真正做到廉洁从政。政府规范性文件、国民经济和社会发展规划、财政预决算、行政审批收费事项、政府采购、重大工程招投标都依法予以公告。要主动接受人大的法律监督、工作监督和政协的民主监督，认真办理人大代表建议、政协委员提案，自觉接受群众监督和舆论监督。

以诚信促优化。打造“诚信九江”，营造“政府诚信、企业守信、人人讲信”的社会信用环境，各级政府要率先垂范，带头讲信用，切实做到承诺有诚、承诺有度、承诺有信，政府抓落实的主要事项向社会公开，年终兑现交帐。引导社会、企业、公民牢固树立“信用立市、信用兴企、信用立人”的理念。提升环境的品牌效应，全面优化政策环境、服务环境、市场环境、法制环境和人文环境，努力营造成本低、回报快、信誉好的投资和创业环境。

以高效促落实。坚持决策科学性与操作坚韧性的高度统一，提高理解上级政策的能力，把握实质、结合实际，创造性地贯彻落实好上级精神。提高具体工作的操作能力，要有敢于承担责任的勇气迎难而上，遇到矛盾不回避、遇到困难不退缩、遇到挫折不气馁。要从群众最关注的热难点入手，从企业最企盼的事情做起，群众有要求的，最小的事也要精心去做；群众反映强烈的，最难的事也要全力去办；群众不满意的，最棘手的事也要痛下决心纠正。

各位代表，在科学发展观指导下，现在全社会高度重视和谐。同心同德是最大的和谐，互勉共为是最实的和谐，同甘共苦是最真的和谐。共同的事业齐心奔，九江的明天一定更美好。让我们高举邓小平理论和“三个代表”重要思想伟大旗帜，紧密团结在以胡锦涛同志为总书记的党中央周围，在省委、省政府和市委的领导下，万众一心，奋发图强，求真务实，开拓进取，圆满完成今年的各项任务，为实现九江在江西率先崛起而努力奋斗！

关联导读：

附件：
